# Supplementary material for: Acceleration-induced spectral beats in strongly driven harmonic oscillators
Source: Nat Commun. 2024 Jul 3;15:5343. doi: 10.1038/s41467-024-49610-5 (PMC11222502; doi:10.1038/s41467-024-49610-5)
Supplement: Supplementary file 1 — Supplementary Information [file 41467_2024_49610_MOESM1_ESM.pdf]

SUPPLEMENTARY INFORMATION:

Acceleration-induced spectral beats in strongly driven harmonic oscillators

A. S. Kuznetsov, K. Biermann, and P. V. Santos

Paul-Drude-Institut für Festkörperelektronik, Leibniz-Institut im Forschungsverbund Berlin e. V.,

Hausvogteiplatz 5-7, 10117 Berlin, German

We present in this document additional material to support and complement the conclusions of the main manuscript.

Supplementary Note 1

The optical setup for the spectroscopic photoluminescence (PL) investigations on polariton traps is displayed in Fig. 1(a). For the conventional PL measurements (i.e., with a spectral resolution of approx. 0.15 meV), the non-resonantly excited emission from the trap is collected by an objective and directed to a spectrometer with CCD detection. This configuration was employed to record the PL spectra displayed in Main text Fig. 1.

As detailed in the Methods section, the high-resolution PL studies were carried out by inserting an etalon in the optical path before the spectrometer. The temperature-stabilized and piezo-tunable Fabry-Perot etalon is fiber-coupled and acts as a tunable spectral filter with a spectral resolution down to 300 MHz and a free spectral range $FSR = 68$ GHz. In the high-resolution measurements, a single-grating spectrometer is employed to discriminate the different spectral orders of the etalon.

Figure 1(b) shows a PL map of the raw spectral data collected using the combination of the etalon and spectrometer. The PL map was acquired by scanning the etalon piezo (voltage V_{piezo}) while keeping the spectrograph wavelength window fixed. The experimental conditions correspond to the ones under high acoustic modulation amplitude displayed in Main text Fig. 3. Here, the energy modulation amplitude covers about 180 GHz corresponding to three etalon FSR's. The different etalon orders as well as

the emission from the ground (GS) and first excited state (ES) are discriminated by the spectrograph, leading to the distinction of the different etalon orders. The dashed yellow and green arrows are included as guides to the evolution of the state energies within two of the etalon orders as they jump between FSRs of the etalon. The spectra displayed in Main text Fig. 3 were obtained by profiling the emission along one of these traces. The integrated PL intensity displayed on the left inset shows the characteristic maxima at the extrema of the modulation amplitude. In the present context, the most important features of the PL map of Fig. 1(b) are the well-defined spectral oscillations near the extrema of the modulation of both the GS and ES. These oscillations correspond to the *acceleration beats* arising from coherent effects induced at high driving amplitudes discussed in detail in the main text.

Supplementary Note 2

In a simplified way, the coupling of acoustic strain to a polariton state can be described via the strain-dependent detuning between bare photon (C) and quantum well (QW) exciton (X) resonances: $\Delta E(\varepsilon) = C(\varepsilon) - X(\varepsilon)$, where ε is the strain field for a particular SAW phase. The individual energies can be written as $X(\varepsilon) = X_0 + a_h \times \varepsilon$ and $C(\varepsilon) = C_0 + \beta \times \varepsilon$, where $a_h = -9$ eV is the GaAs hydrostatic deformation potential, X_0 and C_0 are the unstrained exciton and photon energies. β is the shift of the cavity resonance per unit of strain due to modifications of the spacer thickness and refractive index, which depends on the strain distribution and cavity geometry. In similar microcavities, the ratio of the relative changes of the exciton and photon energies under strain $\Delta X(\varepsilon)/\Delta C(\varepsilon)$ was determined to be in the range from 3 to 20 [1–3]. Therefore, the effect of strain on the photon energy plays a secondary role.

In order to quantitatively describe the light-matter interaction in the MC under the acoustic modulation, we assume that the SAW generates a strain field, which then modulates the bare heavy-hole excitonic resonance of the QWs via the deformation potential (DP) mechanism [1]. With that, we are neglecting SAW-induced radiation pressure effects on the photonic resonances, which in our polariton MCs are normally weaker than

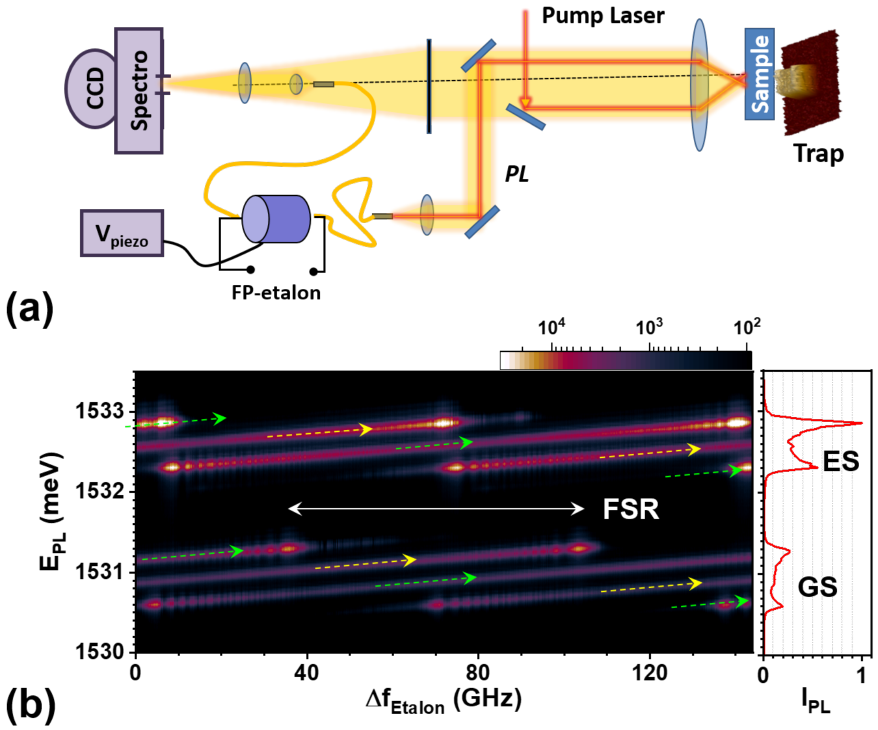

Supplementary Fig. 1. Detection of *acceleration beats* using optical spectroscopy with sub-GHz spectral resolution. (a) Experimental setup for optical spectroscopy on intracavity polariton traps under acoustic excitation. The photoluminescence (PL) non-resonantly excited by the pump laser is collected by an objective and directed to an optical spectrometer (resolution 0.15 meV) with CCD detection. Alternatively, the collection path was blocked and PL was passed through a piezo-driven etalon (controlled by the piezo voltage V_{piezo}) before reaching the spectrometer to perform measurements with high spectral resolution. The etalon has a spectral resolution of 0.3 GHz (1.2 μeV) and a free spectral range of $FSR = 68$ GHz. The spectrometer is used in this case to discriminate the different spectral orders of the etalon. This panel was adapted from Fig. SM5 of the supplementary material of Ref. 4. (b) High-resolution PL spectral map recorded using the etalon in panel (a) showing the emission energy (ΔE_{PL}, vertical scale) and the color-coded emission intensity of the ground (GS) and excited state (ES) of a polariton BEC confined in a 4×4 $\mu\mathrm{m}^2$ intracavity traps under modulation by a SAW excited with nominal rf power $P_{\mathrm{rf}} = 25$ dBm (corresponding to the plots in Fig. 3 of the main text). The horizontal scale is the relative frequency shift Δf_{Etalon} determined by the etalon voltage (V_{etalon}). The dashed green and yellow arrows are guides to the two etalon traces overlapping within the recorded spectral range.

those associated with the excitonic modulation [1]. Furthermore, we will assume that the strain effects are dominated by the hydrostatic component of the SAW strain field, which will be considered to be homogeneous over the trap. This last assumption best applies for a trap with size much smaller than the acoustic wavelength λ_{M} and placed close to an anti-node of the SAW hydrostatic strain field, which is not fully satisfied in the present case with a traps size equal to half λ_{M} [5] (see Sec. 5). Under these considerations, the SAW-induced DP potential commutes with the polariton hamiltonian and, thus, does not mix the unperturbed confined polariton levels. If $s_h(t) = s_{h,0}\cos(\Omega_M t)$ denotes the SAW hydrostatic strain component, the energy of the bare excitonic state [$\phi_X^{(s)}$] will then vary in time according to $\delta_M(t) = a_h s_h(t) = \delta_{M,0}\cos(\Omega_M t)$ around its undisturbed value, which will be taken as the energy reference.

Due to the photonic nature of the confinement, the polariton modes in the trap are associated with bare photonic modes [$\phi_C^{(s)}$] with different confinement energies and, thus, different detunings $\delta_{CX}^{(s)} - \delta_M(t)$ relative to the undisturbed, bare exciton energy, which is taken as the energy reference. Here, $\delta_{CX}^{(s)}$ is the corresponding detuning in the absence of a SAW. The superscript s labels the order of the confined state (i.e., $s =$ GS or ES). The light-matter interaction defining the sth confined polariton state can then be described in the $\{\phi_C, \phi_X\}$ basis by the following perturbation Hamiltonian:

$$\Delta H_{MP}^{(s)}(t) = \begin{pmatrix} \delta_{\mathrm{CX}}^{(s)} - \delta_M(t) & \Omega_R \\ \Omega_R & 0 \end{pmatrix}, \tag{1}$$

where Ω_R is the Rabi light-matter coupling.

We will consider trapped polaritons with a photonic ground state (i.e., with $\delta_{CX}^{(s)} < 0$). For small amplitudes $\chi^{(s)}\hbar\Omega_{\mathrm{M}} \ll \Omega_R$ of the acoustic modulation $\delta_M(t)$, the energy $E_{LP}(t) = \hbar\omega_{LP}(t)$ of the lower polariton eigenstate then becomes:

$$\hbar\omega_{\mathrm{LP}}^{(s)}(t) = \hbar\omega_{\mathrm{LP},0}^{(s)} + \chi^{(s)}\hbar\Omega_{\mathrm{M}}\cos(\Omega_M t) + \chi'^{(s)}\hbar\Omega_{\mathrm{M}}\cos^2(\Omega_M t) \tag{2}$$

with

$$\hbar\omega_{\mathrm{LP},0}^{(s)} = \frac{H_X^2 - 1}{2H_X^2 - 1}\delta_{\mathrm{CX}}^{(s)},$$

$$\hbar\Omega_{\rm M}\chi^{(s)} = H_X^2 \delta_{M,0},$$
$$\hbar\Omega_{\rm M}\chi'^{(s)} = -\frac{\Omega_R^2}{4(\Omega_R^2 + \delta_{\rm CX}^{(p)2})^{3/2}} \delta_{M,0}^2.$$

Here,

$$H_X^2 = \frac{1}{2}\left(1 + \delta_{\rm CX}^{(s)} / \sqrt{\delta_{\rm CX}^{(s)2} + \Omega_R^2}\right) \tag{3}$$

is the (squared) exciton Hopfield coefficient quantifying the relative ratios between the photonic and excitonic contents of the polariton mode.

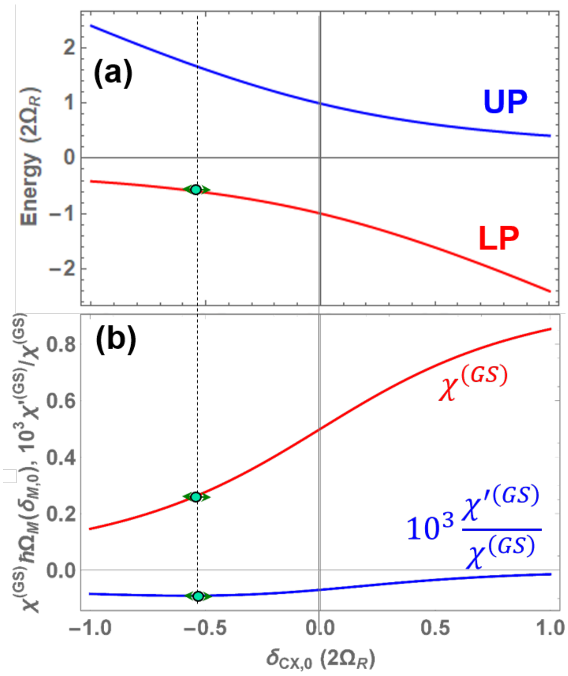

Supplementary Fig. 2. Impact of the cavity-to-exciton detuning on the polariton energy levels and acoustic energy modulation amplitudes. (a) Energy of the lower (LP) and upper polariton (UP) branches as a function of the cavity to exciton detuning, δ_{CX} for the sample used in the studies (Rabi splitting $2\Omega_R = 7.2$ meV). The energies are referenced to the excitonic energy in the absence of acoustic modulation. (b) Modulation coefficients $\chi^{(GS)}$ and $\chi'^{(GS)}$ for the polariton ground state (GS) as a function of detuning. The green filled circle marks the detuning for the sample used in the present studies, the arrows show the variation range of the LP energy induced by the acoustic amplitude of the ground state in Main text Fig. 3.

We determined the modulation coefficients $\hbar\Omega_M\chi^{(s)}$ and $\hbar\Omega_M\chi'^{(s)}$ by solving Eq. (1) using the measured detuning and Rabi coupling for the trap states. Figure 2(a) displays the variation of the lower (LP) and upper polariton (UP) as a function of the static detuning δ_{CX}. The lower panel of the figure shows the dependence of $\chi^{(s)}$ and the ratio $\chi'^{(GS)}/\chi^{(GS)}$ on detuning. The green dot marks the detuning for the intracavity trap used in the experimental studies and the small arrows around it the approximate amplitude of the strain-induced energy modulation for the conditions in Main text Fig. 3 (corresponding to $\chi^{(GS)} = 220$). The quadratic contribution in Eq. (2) for these small modulation amplitudes is much smaller than the linear one. This result justifies neglecting this term in the analysis of the sidebands presented in the main text. We note, however, that the quadratic term is also expected to induce an overall small red-shift of the sideband spectrum by $(1/2)\hbar\Omega_{\mathrm{M}}\chi'^{(s)}$ estimated to be equal to 5 and 4 GHz for the GS and ES, respectively, for $\chi^{(GS)} = 220$. The small magnitude and the approximately equal values for the GS and ES makes it difficult to discriminate these shifts from, e.g., thermal drifts due to the acoustic excitation. The quadratic contribution can also not account for the asymmetric shape of the sideband spectrum for the ES in Main text Fig. 3(b).

The amplitude of the light field emitted by the polaritons can then be obtained from Eq. (2) following the approach described in the main text to yield:

$$\mathscr{E}^{p}_{LP}(t) = \mathscr{E}_{\mathrm{PL},0}[1+\frac{1}{2}\Delta M_{\mathrm{PL}}\sin(\Omega_M t+\psi)]e^{\imath\omega_{\mathrm{PL}}(t)t} \tag{4}$$

$$= \mathscr{E}_{\mathrm{PL},0}[1+\frac{1}{2}\Delta M_{\mathrm{PL}}\sin(\Omega_M t+\psi)]e^{\imath\omega_{\mathrm{PL},0}(t)t} \times \sum_{n=-\infty}^{\infty} J_n(\chi^{(s)})e^{\imath n\Omega_M t} \times \sum_{n=-\infty}^{\infty} J_{2n}(\chi'^{(s)})e^{\imath 2n\Omega_M t} \tag{5}$$

,

where $\mathscr{E}_{\mathrm{PL},0}$ is the undisturbed field amplitude. The prefactor containing ΔM (assumed to be $\ll 1$) accounts for variations in the emission intensity with increasing modulation amplitude. Here, ΔM is the intensity modulation index [6], defined as the maximum excursion of the PL intensity $I_{PL} \propto |\mathscr{E}_{LP}(t)|^2$ during the modulation cycle normalized to

its time-averaged value. When fitting the data in the main text, we verified that the best results were always obtained for $\psi = \pi/2$ and, thus, $\sin(\Omega_M t + \psi) = \cos(\Omega_M t)$, leading to Eq. (6) in the main text.

Figure 3 shows the impact of the amplitude modulation index ΔM on the spectral shape compared to the measured modulation-induced resonance. The parameter values are the same as in Main text Fig. 3(a). ΔM essentially controls the relative amplitude of the sidebands for positive and negative energy shifts relative to the zero-phonon-line at $\Delta f = 0$ without affecting the *acceleration beats*. This occurs because the term involving ΔM in Eq. 5 has a parity with respect to a transformation $n \to -n$ different from the terms associated with $J_n(\chi^{(s)})$ when n is odd. We also checked that the small quadratic contribution proportional to $\chi'^{(s)}$ in Eq. (2) has essentially no effect on the spectral shape.

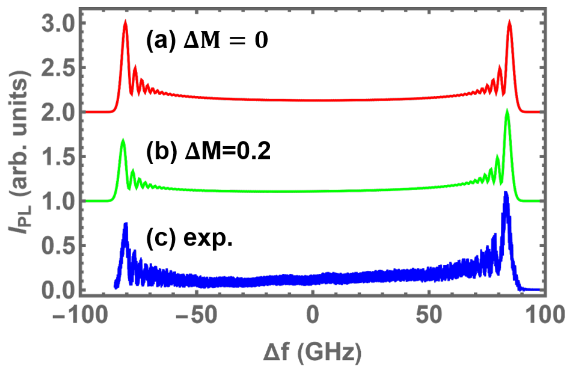

Supplementary Fig. 3. Comparison of the different contributions in Eq. (6) of the main text for the modulated spectrum: (a) $\Delta M = 0.0$, (b) $\Delta M = 0.2$, (c) experimental data of Main text Fig. 3(a).

Supplementary Note 3

Insights into the mechanisms leading to the *acceleration beats* can be gained by calculating the time evolution of the BEC state by neglecting the signal amplitude modulation index (i.e., assuming $\Delta M = 0$) and restricting the energy modulation to the linear term in the first line of Eq. (2) [i. e., by neglecting the second order contribution proportional to $\chi'^{(s)}$]. The time evolution of the states is then obtained by solving:

$$\imath\hbar\frac{\partial\psi^{(s)}}{\partial t} = \left[\hbar\omega^{(s)}_{LP,0} + \chi^{(s)}(\hbar\Omega_M)\cos(\Omega_M t)\right]\psi^{(s)}, \tag{6}$$

where $H_0 = \hbar\omega^{(s)}_{LP,0}$ is the unperturbed, time-independent Hamiltonian. The solution wave function $\psi^{(s)}$ can be expressed as:

$$\psi^{(s)}(t) = \psi^{(s)}(0)e^{-\imath\omega^{(s)}_{LP,0}t}e^{-\imath\chi^{(s)}\sin(\Omega_M t)}. \tag{7}$$

For time intervals Δt close to the extrema of the modulation (i. e., for t around $t_m = (-1)^m\pi/\Omega_M$, with $m = 0, 1, \ldots$), the sinus function in Eq. (7) can be expanded in a power series yielding:

$$\begin{aligned}\psi^{(s)}(t_m+\Delta t) = \psi^{(s)}(0)e^{-\imath\omega^{(s)}_{\mathrm{LP},0}(t_m+\Delta t)} \times \\ \exp\left[-\imath(-1)^m\chi^{(s)}\Delta t\right]\exp\left[+\imath(-1)^m\frac{\chi^{(s)}}{6}(\Omega_M\Delta t)^3\right].\end{aligned} \tag{8}$$

The solid blue and red lines in Fig. 4 display, respectively, the energy modulation and its time derivative. The latter corresponds to the phase factor in Eq. (7).

If we neglect the phase term proportional to $(\Delta t)^3$ in the second line of Eq. (8), the modulation can be approximated by the dashed red line in Fig. 4. The electric field $\mathscr{E}^{(s)}_{LP}(t)$ of the emitted light is proportional to $\psi^{(s)}(t)$. The wavefunction in the reciprocal (i.e., frequency) space (as well as the emitted spectrum) can be obtained via a Fourier transformation of the previous equation:

$$\begin{aligned}\psi^{(s)}(\omega^{(s)}_{\mathrm{LP},0}+\kappa\Omega_M) &= \sum_{m=-\infty}^{+\infty}\int_{\Delta t=-\frac{\pi}{\Omega_M}}^{+\frac{\pi}{\Omega_M}} e^{-\imath\kappa\Omega_M}\psi^{(s)}(t_m+\Delta t)d(\Delta t) \\ &= \psi^{(s)}(0)e^{-\imath\omega^{(s)}_{LP,0}t} \times \\ &\quad \sum_{m=-\infty}^{\infty} e^{-\imath m\Omega_M t} sinc[\pi(\kappa-\chi^{(s)})],\end{aligned} \tag{9}$$

where $sinc(x) = \sin(x)/x$. Since this function is only non-vanishing for $|x| \lesssim 1$, the linear time-dependence of the phase yields angular frequency shifts $\Delta\omega_D = \pm\chi^{(s)}\hbar\Omega_M$ of the spectral line analogous to the Doppler shifts of a source moving with a constant velocity

$c_0\Delta\omega_D/\omega_S$ towards and away from the observer, respectively, where ω_S is the source emission frequency. The sidebands displaced by multiples of Ω_M with respect to this central line appear under the harmonic modulation as a consequence of the emission or absorption of multiple vibrational quanta $\hbar\Omega_M$.

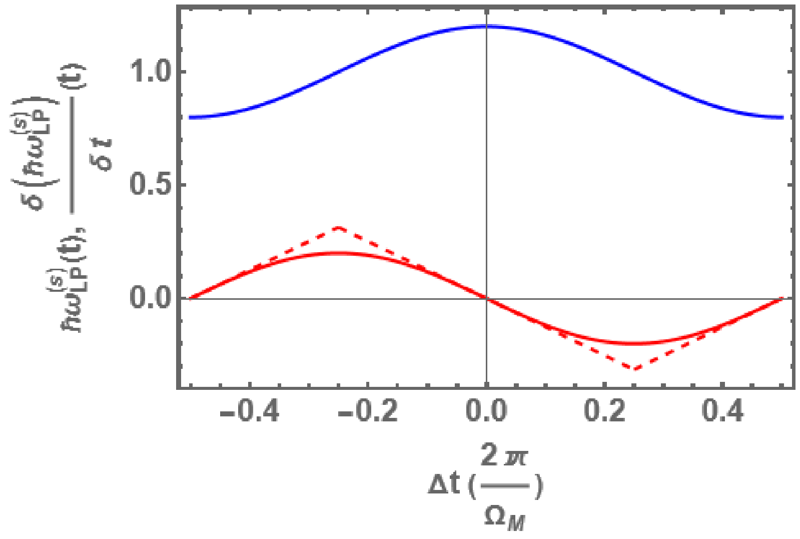

Supplementary Fig. 4. Energy modulation ($\hbar\omega^{(s)}(t)$, blue curve) for the ground state ($s = GS$) and its time derivative within an acoustic period ($\frac{\partial\hbar\omega^{(s)}}{\partial t}(t)$, red curve). The dashed red line displays the linear approximation for the calculation of the Fourier transform described in the text.

We now turn our attention to the complete form of Eq. (8) including the $(\Delta t)^3$ phase term. A closed form Fourier transformation cannot be obtained for this equation. We note, however, that the $(\Delta t)^3$-term induces a phase modulation of the Fourier components $e^{-\imath\Omega_M t}$ with a periodicity in time $\Delta t_c \ll 2\pi/\Omega_M$ satisfying $\frac{\chi^{(s)}}{6}(\Omega_M\Delta t_c)^3 = 2\pi$. This time modulation creates an envelope function for the Fourier components with frequency periodicy $\Delta\omega_c$ given by:

$$\frac{\Delta\omega_c}{\Omega_M} = \frac{\Delta f_c}{f_M} = \left[\frac{2\pi^2}{3}\chi^{(s)}\right]^{1/3}. \tag{10}$$

This equation yields a frequency shift $\Delta f_c = (2\pi)^{-1}\Delta\omega_c$ between the two first *acceleration beats* close to the extrema defined in Main text Fig. 3(b).

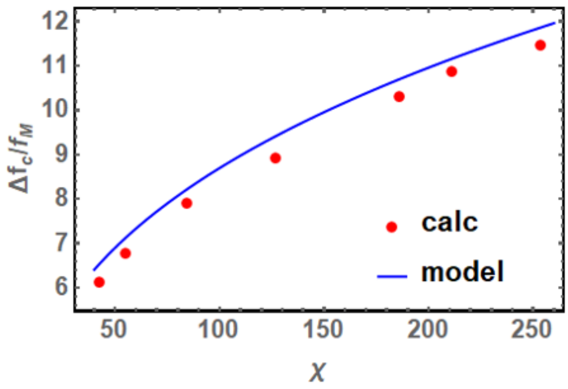

Supplementary Fig. 5. Comparison between the relative frequency spacing $\Delta f_c/f_{\mathrm{M}}$ between the first two *acceleration beats* calculated from Eq. (5) (red dots) with the approximation given by the Eq. (10) (blue curve).

Figure 5 compares the predictions of Eq. (10) (blue curve) with the relative frequency spacing between the first two *acceleration beats* calculated from Eq. (5) assuming $\chi'^{(s)} = 0$ and $\Delta M_{\mathrm{PL}} = 0$ (red dots). The agreement is very good with the predictions of Eq. (10) overestimating the numerical values by less than 4%. The overestimation is attributed to the neglect of higher order terms in the sinus expansion leading to Eq. (8).

Supplementary Note 4

The main challenge for the identification of features due to "acceleration" is to discriminate them from the dominating ones related to linear energy changing rates associated with "velocity" (or Doppler) effects. In the case of harmonic modulation, we show in the manuscript that this can be achieved by increasing the energy modulation index, χ, while preserving the harmonic character of the modulation. In this respect, the use of a high modulation frequency $f_{\mathrm{M}} = \Omega_{\mathrm{M}}/(2\pi)$ is not always helpful since the accompanying high modulation amplitude $\chi\hbar\Omega_{\mathrm{M}}$ may drive the system out of the harmonic regime and introduce significant harmonic distortion, which complicates the identification of acceleration effects.

A few publications have reported modulation procedures yielding a large number of sidebands in different physical systems (see, e.g., Refs. [7–9]). To our knowledge, how-

ever, the conditions for the observation of clear *acceleration beats* were not achieved in any of these reports. As an example, we consider the strain modulation studies reported in Ref. [10], which is one of the few examples involving polaritons. The strain modulation in this case was carried out using very fast strain pulses and is, thus, not harmonic. The most impressive result is the demonstration of non-adiabatic sidebands for modulation in the sub-BEC threshold regime. The modulation is fast enough to yield well-defined sidebands even for polariton coherences corresponding to a linewidth of 0.18 meV, which are significantly larger that those reported here for polariton BECs.

Despite the very fast modulation, the main spectral features are still due to "velocity"-related changes rather than to "acceleration" since the requirements listed above for the observation of *acceleration beats* are not satisfied. To prove this assertion, we made a rough estimate of the relative ratios between the linear and quadratic contributions by approximating the strain modulation (cf. Fig. 1(e) of the main text in Ref. [10]), which consists of only a few (3) sine-like cycles, by a sinusoidal one with the same amplitude (0.37 meV, the maximum lower polariton shift mentioned in the reference) and an effective frequency of 40 GHz (0.16 meV). The latter yields an effective modulation index of only $\chi = 2.3$. The sideband spectrum determined from Eq. (6) in the main text for an harmonic modulation with $\chi = 2.3$ is displayed in Fig. 6: one obtains only three well-defined sidebands spaced by $\hbar\Omega_{\mathrm{M}}$, in good agreement with the experimental results presented in Fig. 2b of the reference paper. These are conventional sidebands related to the "velocity" energy changes expressed by Eq. (9), which take place over a substantial fraction of the harmonic modulation cycle. Here, the red- and blue-shifted sidebands appear, respectively, for positive (i.e., away from) and negative (towards) "velocities" relative to the observer.

The very small number of sidebands in Fig. 6 makes it impossible to see the bunching effects associated with *acceleration beats*, even in the case of a perfect harmonic modulation. In contrast with these results, our data in Main text Fig. 3 was recorded for a comparable energy modulation amplitude (of approx. 0.35 meV) but at a much lower SAW frequency, leading to energy modulation indices χ two order of magnitude larger. In the harmonic regime under long temporal coherences, it is a large energy modulation

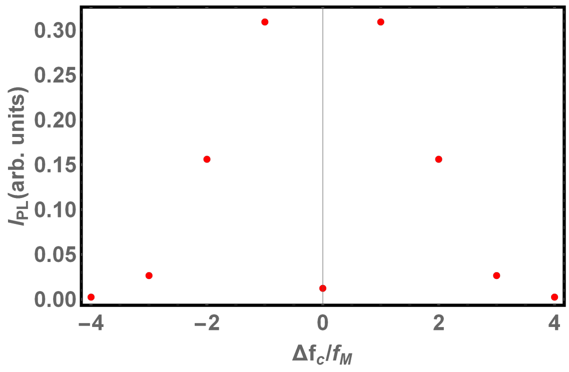

Supplementary Fig. 6. Sideband amplitudes $I_{\rm PL}$ under harmonic modulation with frequency $f_{\rm M} = 40$ GHz and amplitude $\chi h f_{\rm M}$ with $\chi = 2.3$ as determined from Eq. (6) of the main text for the experimental conditions of Ref. 10.

index $\chi = \Delta E/(\hbar\Omega_M)$ that enables pronounced *acceleration beats* rather than just a high modulation frequency ($\hbar\Omega_M$) or amplitude (ΔE).

Finally, the modulation by the SAW in Main text Fig. 3 remains harmonic (and monochromatic) up to very high χ's, which enables the description of the "acceleration effects" using the simple expression in Eq. (6). That is not the case for the pulsed excitation process used in Ref. [10]. As an example, the authors mention a nonlinear decrease in the sideband spacing with strain amplitudes, which they attributed to nonlinear pulse propagation effects.

Supplementary Note 5

The experiments were carried out on a polariton trap placed within an acoustic resonator consisting of two interdigital transducers. The resonator support several longitudinal modes within the frequency emission band of the IDT. These modes are frequency shifted by approximately 0.7 MHz: at the trap location, such a frequency change shift the position of the field anti-nodes by approximately 1 μm, i.e., by 25 % of the trap size. In the present investigations, care was taken to tune the SAW frequency to a mode with anti-node of the SAW hydrostatic strain field aligned with the trap center, as was verified by spatially resolved PL [11]. Under this condition, the SAW strain field has the least

impact on the symmetry of the trap potential and the modulation becomes approximately symmetric for both the ground and excited states. However, we show below that even relatively small shifts of the anti-node with respect to the trap center significantly distorts the trap potential, introducing quadratic contributions that considerably increases the asymmetry, in particular, for the more extended excited states.

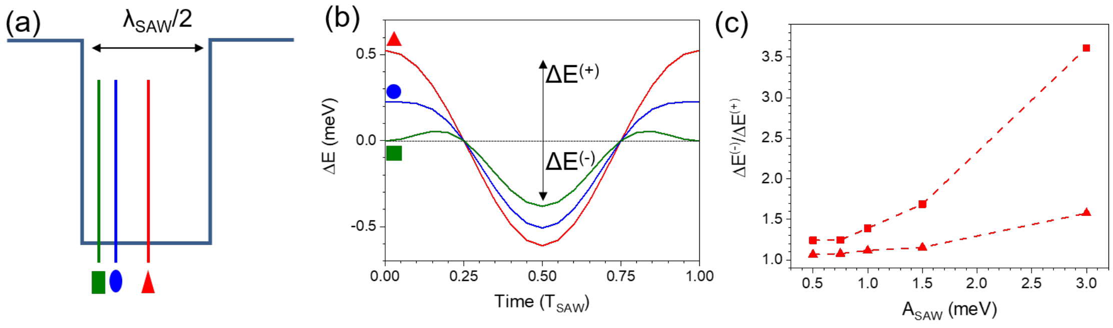

Supplementary Fig. 7. Asymmetric energy shifts during acoustic modulation. (a) Schematic of the simulated configuration of the trap and SAW anti-nodes (vertical lines). (b) Simulated time-evolution of the trap ground state energy for the configurations in (a). λ_M is the SAW wavelength. The energies are referenced to the unmodulated polariton levels. (c) Modulation asymmetry $\Delta E^+/\Delta E^-$, where the energy shifts ΔE^+ and ΔE^- are defined in (b), as extracted from simulated curves akin to the ones in (b) for the ground state (triangles) and the first excited state (squares) for increasing SAW amplitude (A_{SAW}) for the SAW anti-node on the trap center.

We simulated the effects of a shift of the SAW anti-node position on the modulation symmetry using the procedure described in Ref. [11]. Figure 7(a) schematically shows the trap potential with vertical lines designating the simulated positions of the SAW anti-nodes. Figure 7(b) presents the time-evolution of the trap ground state under a SAW for the three anti-node configurations in Fig. 7(a). When the position of the anti-node coincides with the center of the trap, the modulation has the largest amplitude with almost symmetric shifts $\Delta E^{(+)} = \Delta E^{(-)}$ towards higher and lower energies (red curve). However, a small asymmetry remains even for an anti-node position exactly coinciding with the center of the trap: the latter is due to the fact that, due to the different detunings, the strain-induced energy shifts are different for the regions within the trap and in the surrounding barriers. As a consequence, the confinement potential varies during the

acoustic cycle, leading to a non-vanishing energy modulation asymmetry. For the off-center anti-node positions, the energy modulation asymmetry increases and can lead to $\Delta E^{(+)} \ll \Delta E^{(-)}$.

Figure 7(c) shows the modulation asymmetry ($\Delta E^{(-)}/\Delta E^{(+)}$) for the ground and the first excited state with the p-like wave function lobes oriented parallel to the SAW. The calculations were carried out for increasing SAW amplitude (A_{SAW}) assuming SAW anti-node to coincide with the trap center. The asymmetry is much more pronounced for the excited state as compared to the ground state. Based on this figure, we estimate that a shift in the trap position relative to the anti-node of only one μm maintains $\Delta E^{(-)}/\Delta E^{(+)} \sim 1$ for the GS by increases this asymmetry to 1.25 for the measurement conditions in Main text Fig. 3. This value is already larger than the asymmetry of 1.16 of the ES shown in Main text Fig. 3(b).

[1] A. S. Kuznetsov, D. H. O. Machado, K. Biermann, and P. V. Santos, Electrically driven microcavity exciton-polariton optomechanics at 20 GHz, Phys. Rev. X **11**, 021020 (2021), https://arxiv.org/abs/2003.01051.

[2] E. A. Cerda-Méndez *et al.*, Effects of the piezoelectric field in the modulation of exciton-polaritons by surface acoustic waves, Superlattices Microstruct. **49**, 233 (2011).

[3] A. V. Scherbakov *et al.*, Ultrafast control of light emission from a quantum-well semiconductor microcavity using picosecond strain pulses, Phys. Rev. B **78**, 241302 (2008).

[4] A. S. Kuznetsov, K. Biermann, A. A. Reynoso, A. Fainstein, and P. V. Santos, Microcavity phonoritons: a coherent optical-to-microwave interface, Nat. Commun. **14**, 5470 (2023),.

[5] A. S. Kuznetsov, G. Dagvadorj, K. Biermann, M. H. Szymanska, and P. V. Santos, Dynamically tuned arrays of polariton parametric oscillators, Optica **7**, 1673 (2020).

[6] X. Zhu and D. T. Cassidy, Modulation spectroscopy with a semiconductor diode laser by injection-current modulation, J. Opt. Soc. Am. B **14**, 1945 (1997).

[7] V. Torres-Company and A. M. Weiner, Optical frequency comb technology for ultra-broadband radio-frequency photonics, Laser & Photonics Reviews **8**, 368 (2013).

[8] B. Zaks, R. B. Liu, and M. S. Sherwin, Experimental observation of electron–hole recollisions, Nature **483**, 580 (2012).

[9] D. C. Valovcin *et al.*, Optical frequency combs from high-order sideband generation, Optics Express **26**, 29807 (2018).

[10] T. Berstermann *et al.*, Terahertz polariton sidebands generated by ultrafast strain pulses in an optical semiconductor microcavity, Phys. Rev. B **80**, 075301 (2009).

[11] A. S. Kuznetsov, K. Biermann, and P. V. Santos, Dynamic acousto-optical control of confined polariton condensates: From single traps to coupled lattices, Phys. Rev. Research **1**, 023030 (2019).
